# Neolignans from *Nectandra megapotamica* (Lauraceae) Display *in vitro* Cytotoxic Activity and Induce Apoptosis in Leukemia Cells

**DOI:** 10.3390/molecules200712757

**Published:** 2015-07-15

**Authors:** Vitor Ponci, Carlos R. Figueiredo, Mariana H. Massaoka, Camyla F. de Farias, Alisson L. Matsuo, Patricia Sartorelli, João Henrique G. Lago

**Affiliations:** 1 Instituto de Ciências Ambientais, Químicas e Farmacêuticas, Universidade Federal de São Paulo, Diadema, SP 09972-270, Brazil; E-Mails: vitor.ponci@gmail.com (V.P.); psartorelli@unifesp.br (P.S.); 2 Departamento de Microbiologia, Imunologia e Parasitologia, Universidade Federal de São Paulo, São Paulo, SP 04023-062, Brazil; E-Mails: rogernty@hotmail.com (C.R.F.); mari.massa@gmail.com (M.H.M.); camyla.ff@gmail.com (C.F.F.); alisson@gmail.com (A.L.M.)

**Keywords:** *Nectandra megapotamica*, Lauraceae, neolignans, cytotoxic effect, apoptosis

## Abstract

*Nectandra megapotamica* (Spreng.) Mez. (Lauraceae) is a well-known Brazilian medicinal plant that has been used in folk medicine to treat several diseases. In continuation of our ongoing efforts to discover new bioactive natural products from the Brazilian flora, this study describes the identification of cytotoxic compounds from the MeOH extract of *N. megapotamica* (Lauraceae) leaves using bioactivity-guided fractionation. This approach resulted in the isolation and characterization of eight tetrahydrofuran neolignans: calopeptin (**1**), machilin-G (**2**), machilin-I (**3**), aristolignin (**4**), nectandrin A (**5**), veraguensin (**6**), ganschisandrin (**7**), and galgravin (**8**). Different assays were conducted to evaluate their cytotoxic activities and to determine the possible mechanism(s) related to the activity displayed against human leukemia cells. The most active compounds **4**, **5** and **8** gave IC_50_ values of 14.2 ± 0.7, 16.9 ± 0.8 and 16.5 ± 0.8 µg/mL, respectively, against human leukemia (HL-60) tumor cells. Moreover, these compounds induced specific apoptotic hallmarks, such as plasma membrane bleb formation, nuclear DNA condensation, specific chromatin fragmentation, phosphatidyl-serine exposure on the external leaflet of the plasma membrane, cleavage of PARP as well as mitochondrial damage, which as a whole could be related to the intrinsic apoptotic pathway.

## 1. Introduction

The search for chemicals for use in cancer treatments involves many times the discovery of new prototypes based in natural products, especially those obtained from plants and microorganisms [1]. This approach has led to the discovery of several drugs currently used in cancer therapy, including vinblastine (Velban^®^), vincristine (Oncovin^®^), vindesine (Eldisine^®^), vinorelbine (Navelbine^®^), paclitaxel (Taxol^®^), docetaxel (Taxotere^®^), podofilotoxin, etoposide (Etopophos^®^), teniposide (Vumon^®^), camptothecin, topotecan (Hycamtin^®^), and irinotecan (Camptosar^®^) [2,3,4]. However, considering that less than 2% of all plants with therapeutic properties have been properly analyzed for the detection and isolation of compounds with cytotoxic activity [5], there is an extraordinary potential to discover new compounds with antitumor activity in Brazilian plant species due to their high phylogenetic and chemical diversity. In this context, *Nectandra megapotamica* (Lauraceae), popularly known in Brazil as “canela-lora”, “canela-preta” or “canela-do-mato”, has been used in traditional medicine in the treatment of rheumatism and to relieve pain [6,7]. Phytochemically, this plant accumulates several natural products such as neolignans, phenylpropanoids and alkaloids with antileishmanial [8], anti-inflammatory [9] and antitrypanosomal [7] activities. As part of a continuous effort to find new cytotoxic compounds from Brazilian flora [10,11], this study reports a bioactivity-guided fractionation of the MeOH extract from the leaves of *N. megapotamica*, aiming at the isolation of compounds with *in vitro* cytotoxic activity. Additionally, we also propose a possible mechanism of action for the most active compounds against human leukemia (HL-60) cells.

## 2. Results and Discussion

**Figure 1:**
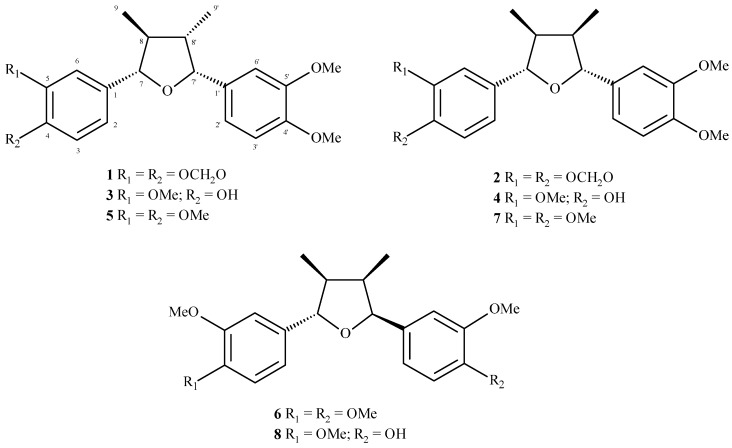
Tetrahydrofuran neolignans **1**–**8** isolated from the leaves of *N. megapotamica*.

After several chromatographic procedures, guided by the evaluation of cytotoxic activity, we isolated eight related compounds from MeOH extract from leaves of *N. megapotamica*. The ^1^H-NMR spectra of **1**–**8** indicated the presence of a 1,3,4-trisubstituted aromatic ring due to the signals ranging from δ 6.7 to 7.1 (H-2/H-2′, H-3/H-3′ and H-6/H-6′). Additionally, signals attributed to methoxyl or methylenedioxyl groups were detected at approximately δ 4.0 (s) and δ 6.0 (s), respectively. These data, associated to the doublets assigned to oxymethine hydrogens H-7/H-7′ (δ 4.43–5.48) as well as to methyl groups linked to C-8/C-8′ (δ 0.63–1.08), suggested the occurrence of tetrahydrofuran neolignans [12]. The ^13^C-NMR of compounds **1**–**8** showed peaks at δ 108.1–149.5 attributed to aromatic rings (C-1 to C-6 and C-1′ to C-6′), a tetrahydrofuran unit at δ 43.2–47.4 (C-8/C-8′) and δ 85.1–88.2 (C-7/C-7′) as well as methyl groups (C-9/C-9′) at δ 8.9–14.8. Substituents on the aromatic rings such as methoxyl and/or methylenedioxyl groups were observed approximately at δ 56 and δ 101, respectively. Finally, comparison of spectral data with those reported in the literature [12,13,14,15,16,17], associated to LREIMS analysis, allowed the identification of calopeptin (**1**), machilin-G (**2**), machilin-I (**3**), aristolignin (**4**), nectandrin A (**5**), veraguensin (**6**), ganschisandrin (**7**), and galgravin (**8**), whose structures are shown in Figure 1. 

As previously reported in the literature, *N. megapotamica* produces different compounds with important pharmacological and biological activities such as trypanocidal alkaloids [6], fungitoxic phenylpropanoids [18] as well as anti-inflammatory, trypanocidal and antileishmanial neolignans [7,8]. However, there are no previous studies evaluating the cytotoxic activity against cancer cells of crude extract and/or isolated compounds. Thus, cytotoxicity of crude MeOH extract, partition phases and compounds **1**–**8** obtained from leaves of *N. megapotamica* was determined on murine melanoma (B16F10) and human (HeLa, MCF7, A2058 and HL-60) cancer cell lines. Since crude MeOH extract displayed activity against all tested cell lines, this material was partitioned using hexane, CH_2_Cl_2_ and EtOAc. Hexane and CH_2_Cl_2_ phases showed activity while EtOAc phase was inactive (IC_50_ > 100 µg/mL) (Table 1).

**Table 1:** IC_50_ (µg/mL) values of neolignans **1**–**8** from *N. megapotamica* and positive control (cisplatin) against different tumor cell lineages.

	IC_50_ (µg/mL)				
	Cancer Cell Line				
	*B16F10*	*HeLa*	*MCF7*	*A2058*	*HL-60*
**MeOH extract**	75.1 ± 2.9	83.1 ± 4.0	93.3 ± 2.5	75.2 ± 5.7	56.1 ± 5.8
**Hexane phase**	81.1 ± 1.8	>100	>100	>100	89.0 ± 4.2
**CH_2_Cl_2_ phase**	57.3 ± 3.1	80.6 ± 2.4	73.2 ± 1.6	78.1 ± 3.2	39.3 ± 3.4
**EtOAc phase**	>100	>100	>100	>100	>100
**1**	72.8 ± 3.0	>100	>100	98.1 ± 1.2	73.1 ± 4.9
**2**	>100	>100	>100	>100	89.0 ± 2.1
**3**	>100	>100	>100	>100	53.5 ± 3.6
**4**	41.1 ± 2.4	65.1 ± 2.8	54.3 ± 2.8	67.8 ± 3.4	14.2 ± 0.7
**5**	40.4 ± 1.9	55.4 ± 2.7	69.0 ± 3.3	59.7 ± 3.1	16.9 ± 0.8
**6**	37.9 ± 1.7	>100	>100	>100	18.8 ± 0.4
**7**	31.2 ± 1.3	99.1 ± 5.2	>100	75.0 ± 3.9	29.1 ± 1.2
**8**	60.0 ± 3.2	68.0 ± 3.2	>100	>100	16.5 ± 0.8
**cisplatin**	53.1 ± 4.2	20.6 ± 1.5	21.1 ± 1.4	43.2 ± 3.2	21.2 ± 2.3

Following bioactivity-guided fractionation, individual compounds were isolated from different fractions and their IC_50_ values were determined against different tumor cell lines (Table 1). Compounds **1** and **2** were isolated from the hexane phase, being **1** the most active. IC_50_ values for human cancer cells varied from 73.1 ± 4.9 to 98.1 ± 1.2 µg/mL, while the IC_50_ for murine melanoma B16F10 was 72.8 ± 3.0 µg/mL. Using the same approach, the CH_2_Cl_2_ fraction was also subjected to chromatographic separation and compounds **3**–**8** were isolated from the bioactive fractions. Compounds **3** and **7** displayed weak cytotoxic activity against human cancer cells. On the other hand, compounds **4** and **5** displayed activity against all tested cell lines with IC_50_ values ranging from 69.0 ± 3.3 to 14.2 ± 0.7 µg/mL. Compound **6** showed strong activity against HL-60 (IC_50_ of 18.8 ± 0.4 µg/mL) and B16F10 (IC_50_ 37.9 ± 1.7 µg/mL) cell lines, higher than the positive control drug, cisplatin (IC_50_ of 21.2 ± 2.3 and 53.1 ± 4.2 µg/mL, respectively), but was inactive against the other tested human cancer cells. Similarly, compound **8** displayed promising cytotoxic activity to HL-60 cell lines (IC_50_ 16.5 ± 0.8 µg/mL) but lower activity against other human cancer cells. Considering the structural differences among the isolated neolignans, it was suggested that the presence of hydroxyl and methoxyl substituents on the aromatic rings of **4** and **8** might have contributed to the higher efficacy among the tested compounds. However, if these groups are substituted by a methylenedioxyl moiety, the activity is strongly reduced, as could be observed to compounds **1** and **2**. Otherwise, the presence of four methoxyl groups, as observed to compounds **4**–**6** and **8**, caused an increment in the cytotoxic potential.

Additionally, because compounds **4**, **5** and **8** displayed higher antitumor potential against HL-60 cells (human leukemia), further investigation for better understanding their cytotoxic properties was carried out. Treatment of HL-60 cells with compounds **4**, **5** and **8** induced several apoptotic hallmarks, such as cytoplasm retraction, bleb formation (apoptotic bodies) and condensation of nuclear material (pyknosis) followed by chromatin fragmentation (karyorrhexis) [19,20]. To verify whether morphological alterations induced by **4**, **5** and **8** were accompanied by changes in the mitochondrial transmembrane potential (ΔΨm), the cationic lipophilic dye, TMRE, was used. It has been reported that ΔΨm collapse constitutes an early event of apoptosis and marks an already irreversible stage of the apoptotic process [21,22,23].

**Figure 2:**
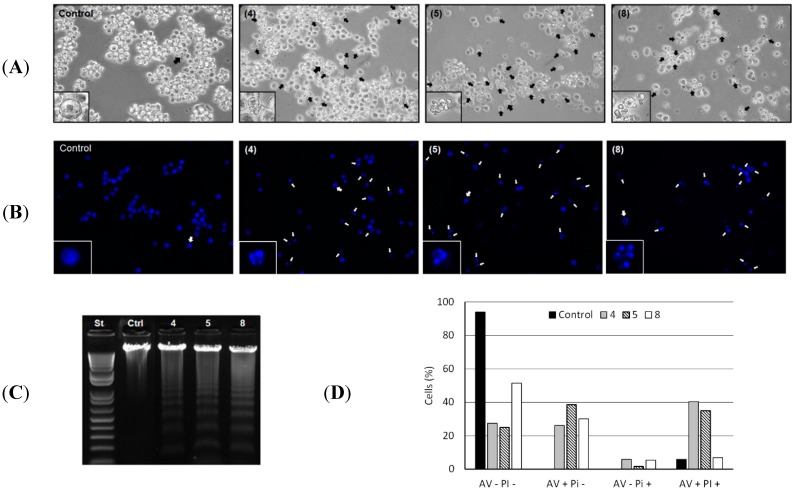
(**A**) Morphological changes induced by **4**, **5** and **8**. HL-60 cells were incubated with 50 µg/mL of each compound for 24 h and analyzed by light microscopy. The formation of blebs in cell membrane was evidenced in all HL-60 treated cells. Magnification ×400; (**B**) Chromatin condensation and fragmentation analysis by fluorescence microscopy. 1 × 10^5^ HL-60 cells were incubated with 50 µg/mL of **4**, **5** and **8** for 24 h and stained with DAPI (blue) for chromatin analysis (original magnification, ×400). White arrows indicate chromatin condensation and fragmentation process; (**C**) DNA analysis of HL-60 cells after incubation with 50 µg/mL of **4**, **5** and **8** for 24 h. DNA was extracted from tumor cells and analyzed in 1% agarose gel; (**D**) Translocation of phosphatidylserine in B16F10-Nex2 (5 × 10^5^ cells) previously incubated with compounds **4**, **5** and **8** at 100 µg/mL and negative control (RPMI medium) for 24 h. AV−PI− (live cells); AV+PI− (early apoptotic cells); AV−PI+ (necrotic cells); AV+PI+ (late apoptotic cells).

Morphological alterations in HL-60 cells induced by the compounds **4**, **5** and **8** are shown in Figure 2A. The formation of blebs and cell fragmentation could be directly related to an apoptosis process as described elsewhere [19]. Chromatin condensation and fragmentation were also evidenced. Analysis by fluorescence microscopy of the genome integrity of HL-60 cells revealed chromatin condensation in 60.2%, 67.8% and 37.0% of total cells treated with 50 µg/mL of **4**, **5** and **8**, respectively (Figure 2B). Cleavage of chromosomal DNA into oligonucleosomal fragments was alternatively evaluated by gel electrophoresis of the tumor cell DNA after incubation with 50 µg/mL of each compound for 24 h, resulting in a ladder fragmentation pattern observed in agarose gel (Figure 2C).

The externalization of the inward-facing phosphatidylserine of the cell membrane to the outer layer is a well-known apoptotic hallmark, and Annexin V is a phosphatidylserine-binding protein currently used for the detection of apoptosis [19]. Thus, induction of apoptosis by compounds **4**, **5** and **8** in HL-60 cells was further confirmed by evaluation of phosphatidylserine externalization using the Annexin-V/PI assay. We observed that compounds **4**, **5** and **8** significantly increased the number of early apoptotic cells (AV+/PI−: 26.2%, 38.5% and 12.7%) and late apoptotic cells (AV+/PI+: 34.5%, 29% and 1%) relative to negative control (Figure 2D). Early apoptosis is characterized by single Annexin V (AV) staining and double staining of AV and PI only occurs in the later stages of apoptosis, probably when cell membranes have been damaged during apoptosis or in necrotic cells with no single AV staining [24]. The necroptosis cell death process was discarded during treatment with compounds **4**, **5** and **8** since their cytotoxic activity at 100 µg/mL were not inhibited by necrostatin-1 (data not shown), a well-known necroptosis inhibitor [25].

**Figure 3:**
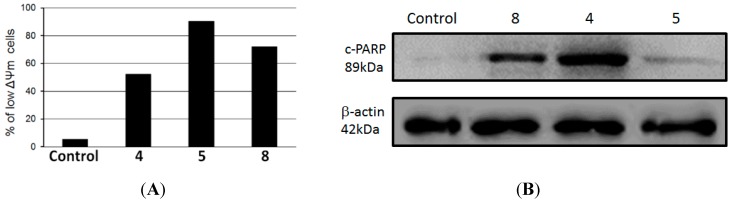
(**A**) Mitochondrial membrane depolarization induced by compounds **4**, **5** and **8** in HL-60 cells; (**B**) Lysates from HL-60 cells, previously treated with compounds **4**, **5** and **8** at 100 µg/mL for 24 h at 37 °C, were analyzed by Western blotting. Antibodies against cleavage PARP and β-actin (protein loading control) were used.

Apoptosis occur mainly by the extrinsic, or death receptor pathway, and the intrinsic, or mitochondrial, pathway [26]. Since HL-60 cells undergo apoptosis during compounds **4**, **5** and **8** treatment, we investigated whether the intrinsic mitochondrial pathway might be involved. Cells were treated with 100 µg/mL of **4**, **5** and **8** for 24 h and then ΔΨm was determined by flow cytometry using TMRE probe. As determined by flow cytometry, compounds **4**, **5** and **8** induced loss of mitochondrial membrane potential in 90.4%, 72.2% and 52.2% of HL-60 treated cells, respectively, whereas only 5.3% of untreated control cells presented a low ΔΨm (Figure 3A). In addition, we observed that there was an increase in the levels of cleavage of poly(ADP-ribose) polymerase (PARP) in HL-60 treated cells compared to a negative control, as observed by immunoblotting positive spots at 89 kDa (Figure 3B), a well-known apoptotic hallmark in HL-60 cells [24]. Cleavage of PARP is catalyzed by caspase-3 in the later events of apoptosis and is related to depletion of NAD and ATP during apotosis [27].

Based on the obtained data, we believe that these compounds might trigger cell death through the mitochondria-mediated apoptosis pathway in HL-60 cells. Current available chemotherapic treatments of leukemia have many side effects and deficiencies, including drug-resistance against apoptosis [28,29]. Therefore, the identification of novel compounds that have potential to seed new chemotherapic drugs that specifically act against this type of cancer is of utmost importance. 

## 3. Experimental Section

### 3.1. General Experimental Procedures

^1^H- and ^13^C-NMR spectra were recorded, respectively, at 300 and 75 MHz on an Avance III 300 spectrometer (Bruker, Fremont, CA, USA). CD_3_OD or CDCl_3_ (Aldrich, St. Louis, MO, USA) were used as solvent and as internal standard. LREIMS (70 eV) were measured on a 14B/QP5050A spectrometer (Shimadzu, Kyoto, Japan). Silica gel (Merck, Kenilworth, NJ, USA, 230–400 mesh) and Sephadex LH-20 (Aldrich) were used for column chromatography separations while silica gel 60 PF_254_ (Merck) was used for analytical TLC. Semi-preparative HPLC chromatography separations were performed on an Ultimate 3000 system (Dionex, Sunnyvale, CA, USA) equipped with a quaternary pump system, a PDA detector, and a Phenomenex reversed-phase C_18_ column (250 × 10.0 mm, 5 μm). All solvents used for column chromatography were of analytical grade (CAAL, São Paulo, Brazil) while those used for HPLC separations were of HPLC grade (Tedia, Fairfield, OH, USA).

### 3.2. Plant Material

Leaves of *N. megapotamica* were collected at the Atlantic Forest area, São Paulo State, Brazil, in April, 2010. The identification was performed by Dr. Maria Claudia M. Young (Instituto de Botânica-SP) and a voucher specimen has been deposited in the Herbarium of Instituto de Botânica de São Paulo.

### 3.3. Extraction and Isolation

Dried leaves of *N. megapotamica* (580 g) were powdered and exhaustively extracted with MeOH (5 × 1000 mL at room temperature). The obtained material was evaporated to dryness under reduced pressure, resuspended in 3:1 MeOH-H_2_O and subjected to sequential partition using hexane, CH_2_Cl_2_ and EtOAc. After evaporation of solvent under reduced pressure, hexane (28 g), CH_2_Cl_2_ (3 g) and EtOAc (7 g) phases were obtained. Part of the active hexane phase (27 g) was subjected to CC over SiO_2_ eluted with increasing amounts of EtOAc in hexane and MeOH in EtOAc. This procedure afforded eight fractions (H1-H8), in which activity was detected at fraction H5 (1.5 g). Part of this fraction (15 mg) was purified by HPLC (RP-18, MeOH:H_2_O 75:25, flow 1.75 mL/min) to afford **1** (4 mg) and **2** (4 mg). Part of the active CH_2_Cl_2_ phase (1.7 g) was fractioned over Sephadex LH-20 eluted with MeOH to give four fractions (D1-D4). As the cytotoxicity was detected on the group D2 (857 mg), this material was subjected to CC over SiO_2_ using increasing amounts of EtOAc in hexane as eluent, to afford eight fractions (D2-1 to D2-8). Part of the active fraction D2-4 (75 mg) was purified by HPLC (RP-18, MeOH:H_2_O 75:25, flow 2.0 mL/min) to afford **3** (4 mg), **4** (26 mg), **5** (13 mg), **6** (11 mg), **7** (3 mg), and **8** (2 mg). 

### 3.4. Cell Lines

Murine melanoma cell line B16F10 as well as human melanoma A2058, breast adenocarcinoma MCF7, human leukemia HL-60, and human cervical carcinoma cell line HeLa were provided by the Experimental Oncology Unit (UNONEX) of the Federal University of São Paulo (UNIFESP, São Paulo SP, Brazil). Cells were cultured at 37 °C in a humidified atmosphere containing 5% CO_2_, in RPMI 1640 medium (Invitrogen, Carlsbad, CA, USA) supplemented with 10 mM *N*-2-hydroxyethylpiperazine-*N*-2-ethanesulfonic acid (Hepes, Sigma, St. Louis, MO, USA), 24 mM sodium bicarbonate (Sigma), 40 mg/L gentamycin (Schering-Plough, São Paulo, Brazil), pH 7.2, and 10% fetal calf serum (Invitrogen).

### 3.5. In Vitro Cytotoxicity Assay

Crude MeOH extract, partition phases and compounds **1**–**8** were suspended in dimethylsulfoxide (DMSO) at a final concentration of 10 mg/mL, and finally diluted in complete RPMI medium supplemented with 10% fetal calf serum. Crude extracts and partition phases were evaluated at 300 µg/mL while compounds **1**–**8** were assayed using different concentrations, ranging from 0 to 100 μg/mL. These materials were incubated with 1 × 10^4^ cells in a 96-well plate at 37 °C and 5% CO_2_. After 24 h of incubation, cell viability was assessed using the Cell Proliferation Kit I (MTT, Sigma), a MTT-based colorimetric assay as previously described [30,31]. Readings were made in a plate reader (Spectra Max M2e, Molecular Devices, Sunnyvale, CA, USA) at 570 nm with a reference of 650 nm. All experiments were performed in triplicates using cisplatin (Sigma) and DMSO 1% as positive and negative controls, respectively.

### 3.6. Morphology, Chromatin Condensation and Fragmentation Analysis

Morphology, chromatin condensation and fragmentation were analyzed by fluorescence microscopy. HL-60 cells (1 × 10^4^) were seeded on 96-well plates and incubated with 50 µg/mL of compounds **4**, **5** and **8** during 24 h at 37 °C and 5% CO_2_. For morphology analysis, images were processed using a light inverted microscope (Magnification ×400). To analyze the condensation of chromatin, cells were harvested, washed in PBS and fixed for 15 min at room temperature using MeOH. Cells were pelleted and stained with 10 µg/mL DAPI (Sigma) in PBS for 15 min. The cells were analyzed by fluorescence microscopy using an inverted fluorescence microscope (Eclipse TS100, magnification ×40, Nikon, Tokyo, Japan). Alternatively, DNA fragmentation was assessed by electrophoresis in a 1% agarose gel. HL-60 (1 × 10^5^) cells were incubated with 50 µg/mL of compounds **4**, **5** and **8** for 24 h and then total DNA was extracted, processed and analyzed as previously described [32].

### 3.7. Detection of Mitochondrial Membrane Potential (ΔΨm)

The cationic lipophilic dye tetramethylrhodamine ethyl ester (TMRE) was used to determine the mitochondrial membrane potential. 5 × 10^5^ cells (HL-60) were grown in a 12-well culture plate and incubated with 100 µg/mL of **4**, **5** and **8** for 24 h at 37 °C. Subsequently, cells were processed and analyzed as previously described [33].

### 3.8. Annexin V and Propidium Iodide Labeling

Annexin V (AV) positive HL-60 cells were detected using Annexin V-FITC Apoptosis kit (Sigma-Aldrich, St. Louis, MO, USA). 5 × 10^5^ tumor cells were cultured in 6-well plates and further incubated with 100 µg/mL of compounds **4**, **5** and **8** or complete medium (negative control) for 24 h at 37 °C. After treatment period, cells were washed three times with PBS and incubated with 1X binding buffer. Cells were incubated with binding buffer (10 mM HEPES/NaOH, pH 7.5, 140 mM NaCl and 2.5 mM CaCl_2_) in presence of 5 µL of AV-FITC and 10 µL of propidium iodide (PI) for 10 min at room temperature and immediately analyzed by flow cytometry (BD Bioscience FACSCanto II equipment, Franklin Lakes, NJ, USA). Quantification of live cells (AV−/PI−), necrotic cells (AV−/PI+), early apoptotic cells (AV+/PI−) and late apoptotic cells (AV+/PI+), were performed using FlowJo software (version 9.5.3, Tree Star Inc., Ashland, OR, USA).

### 3.9. Cell Lysate Extracts and Western Blotting

For protein extraction, 5 × 10^5^ HL-60 cells, previously incubated with 100 µg/mL of compounds **4**, **5**, **8** and negative control for 24 h, were washed in PBS and lysed by adding 100 µL of 1X SDS sample buffer (62.5 mM Tris-HCl, pH 6.8 at 25 °C, 2% *w*/*v* SDS, 10% glycerol, 50 mM DTT, 0.01% *w*/*v* bromophenol blue) in the presence of phosphatase and protease inhibitors and heated to 95 °C for 5 min. Total proteins from each cell lysate were separated in SDS gel electrophoresis and Western blotting was carried out as described elsewhere [33], and rabbit anti cleavaged PARP (c-PARP) was used. Anti β-actin was used as loading control. Both antibodies were purchased from Cell Signaling Technology (Beverly, MA, USA). Secondary antibody conjugated with IgG horseradish peroxidase was purchased from Sigma-Aldrich. Immunoreactivity was detected using the Immobilon solution (Millipore, Billerica, MA, USA) in an Uvitec Alliance 2.7 instrument (Cambridge, UK).

### 3.10. Statistical Analysis

The obtained data represent the means and standard deviations from three independent experiments. The IC_50_ were estimated using correlation curves carried out in Origin 5.0 statistical software (OriginLab, Northampton, MA, USA) for Windows and differences among them were assayed using Student’s *t*-test (* *p* < 0.05 *vs* control conditions).

## 4. Conclusions

In conclusion, eight tetrahydrofuran neolignans **1**–**8** were isolated from leaves of *N. megapotamica* and identified by comparison to previously reported NMR and LREIMS data in the literature. The isolated compounds displayed cytotoxic activity against different cell lineages being **4**, **5** and **8** the most active against human leukemia tumor cells (HL-60). Due to these results, an investigation of the possible mechanism involved in cell death was conducted. The obtained data showed that compounds **4**, **5** and **8** induced specific apoptotic hallmarks, such as plasma membrane bleb formation, together with nuclear DNA condensation, specific chromatin fragmentation, and mitochondrial damage, which may be related to the intrinsic apoptotic pathway. Therefore, since there is a great need for the development of novel anticancer prototypes for the treatment of leukemia, tetrahydrofuran neolignans should be considered as a model to future drug design, given their great ability to break down tumor cell resistance to apoptosis.

## References

[1] Gordaliza M. (2007). Natural products as leads to anticancer drugs. Clin. Transl. Oncol..

[2] Gosttesman M.M. (2002). Mechanisms of cancer drug resistance. Ann. Rev. Med..

[3] Khazir J., Riley D.L., Pilcher L.A., De-Maayer P., Mir B.A. (2014). Anticancer agents from diverse natural sources. Nat. Prod. Commun..

[4] Kinghorn A.D., Chin Y.W., Swanson S.M. (2009). Discovery of natural product anticancer agents from biodiverse organisms. Curr. Opin. Drug. Discov. Dev..

[5] Zhang J.T. (2002). New drugs derived from medicinal plants. Therapie.

[6] Santos-Filho D., Gilbert B. (1975). The alkaloids of *Nectandra megapotamica*. Phytochemistry.

[7] Silva-Filho A.A., Albuquerque S., Silva M.L., Eberlin M.N., Tomazela D.M., Bastos J.K. (2004). Tetrahydrofuran lignans from *Nectandra megapotamica* with trypanocidal activity. J. Nat. Prod..

[8] Silva-Filho A.A., Costa E.S., Cunha W.R., Silva M.L., Nanayakkara N.P., Bastos J.K. (2008). *In vitro* antileishmanial and antimalarial activities of tetrahydrofuran lignans isolated from *Nectandra megapotamica* (Lauraceae). Phytother. Res..

[9] Souza G.H.B., Silva-Filho A.A., Souza V.A. (2004). Analgesic and anti-inflammatory activities evaluation of (−)-*O*-acetyl, (−)-*O*-methyl, (−)-*O*-dimethylethylaminecubebin and their preparation from (−)-cubebin. II Farm..

[10] Capello T.M., Martins E.G.A., Farias C.F., Figueiredo C.R., Matsuo A.L., Passero L.F.D., Oliveira-Silva D., Sartorelli P., Lago J.H.G. (2015). Chemical composition and *in vitro* cytotoxic and antileishmanial activities of extract and essential oil from leaves of *Piper cernuum* Vell. (Piperaceae). Nat. Prod. Commun..

[11] Grecco S.S., Martins E.G.A., Girola N., Figueiredo C.R., Matsuo A.L., Soares M.G., Bertoldo B.C., Sartorelli P., Lago J.H.G. (2015). Chemical composition and *in vitro* cytotoxic effects of the essential oil from *Nectandra leucantha* leaves. Pharm. Biol..

[12] Wang L., Zhou X., Xu T., Yang X., Liu Y. (2012). Lignans from *Saurus chinensis*. Chem. Nat. Comp..

[13] Barata L.S., Baker P.M., Gottlieb O.R., Ruveda E.A. (1975). Neolignans of *Virola surinamensis*. Phytochemistry.

[14] Doskotch R.W., Flom M.S. (1972). Acuminatin, a new bis-phenylpropide from *Magnolia acuminata* L. Tetrahedron.

[15] Yue J.M., Chen Y.Z., Hua S.M., Cheng J.L., Cui Y.X. (1989). Ganschisandrine, a lignan from *Schisandra sphenanthera*. Phytochemistry.

[16] Lopes N.P., Blumenthal E.E.A., Cavalheiro A.J., Kato M.J., Yoshida M. (1996). Lignans, γ-lactones and propiophenones of *Virola surinamensis*. Phytochemistry.

[17] Shimomura H., Sashida Y., Oohara M. (1988). Lignans from *Machilus thumbergii*. Phytochemistry.

[18] Garcez F.R., Garcez W.S., Hamerski L., Miguita C.H. (2009). Phenylpropanoids and other bioactive constituents from *Nectandra megapotamica*. Quim. Nova.

[19] Kroemer G., Galluzzi L., Vandenabeele P., Abrams J., Alnemri E.S., Baehrecke E.H., Blagosklonny M.V., El-Deiry W.S., Golstein P., Green D.R. (2009). Classification of cell death: Recommendations of the Nomenclature Committee on Cell Death 2009. Cell Death Differ..

[20] Galluzzi L., Vitale I., Abrams J.M., Alnemri E.S., Baehrecke E.H., Blagosklonny M.V., Dawson T.M., Dawson V.L., El-Deiry W.S., Fulda S. (2012). Molecular definitions of cell death subroutines: Recommendations of the Nomenclature Committee on Cell Death 2012. Cell Death Differ..

[21] Kroemer G., Dallaporta B., Resche-Rigon M. (1998). The mitochondrial death/life regulator in apoptosis and necrosis. Ann. Rev. Physiol..

[22] Lago J., Santaclara F., Vieites J.M., Cabado A.G. (2005). Collapse of mitochondrial membrane potential and caspases activation are early events in okadaic acid-treated Caco-2 cells. Toxicon.

[23] Ly J.D., Grubb D.R., Lawen A. (2003). The mitochondrial membrane potential (deltapsi(m)) in apoptosis: An update. Apoptosis.

[24] Koopman G., Reutelingsperger C.P., Kuijten G.A., Keehnen R.M., Pals S.T., Van-Oers M.H. (1994). Annexin V for flow cytometric detection of phosphatidylserine expression on B cells undergoing apoptosis. Blood.

[25] Vandenabeele P., Grootjans S., Callewaert N., Takahashi N. (2013). Necrostatin-1 blocks both RIPK1 and IDO: Consequences for the study of cell death in experimental disease models. Cell Death Differ..

[26] Elmore S. (2007). Apoptosis: A review of programmed cell death. Toxicol. Pathol..

[27] Boulares A.H., Yakovlev A.G., Ivanova V., Stoica B.A., Wang G., Iyer S., Smulson M. (1999). Role of poly(ADP-ribose) polymerase (PARP) cleavage in apoptosis. Caspase 3-resistant PARP mutant increases rates of apoptosis in transfected cells. J. Biol. Chem..

[28] Mims A., Stuart R.K. (2013). Developmental therapeutics in acute myelogenous leukemia: Are there any new effective cytotoxic chemotherapeutic agents out there?. Curr. Hematol. Malign..

[29] Evan G.I., Vousden K.H. (2001). Proliferation, cell cycle and apoptosis in cancer. Nature.

[30] Santana J.S., Sartorelli P., Guadagnin R.C., Matsuo A.L., Figueiredo C.R., Soares M.G., Silva A.M., Lago J.H.G. (2012). Essential oils from *Schinus terebinthifolius* leaves chemical composition and *in vitro* cytotoxicity evaluation. Pharm. Biol..

[31] Figueiredo C.R., Matsuo A.L., Massaoka M.H., Polonelli L., Travassos L.R. (2014). Anti-tumor activities of peptides corresponding to conserved complementary determining regions from different immuno globulins. Peptides.

[32] Matsuo A.L., Figueiredo C.R., Arruda D.C., Pereira F.V., Scutti J.A., Massaoka M.H., Travassos L.R., Sartorelli P., Lago J.H.G. (2011). Antimetastatic and apoptotic effect induced by α-pinene isolated from *Schinus terebinthifolius* Raddi. Biochem. Biophys. Res. Commun..

[33] Massaoka M.H., Matsuo A.L., Figueiredo C.R., Farias C.F., Girola N., Arruda D.C., Scutti J.A., Romoff P., Favero O.A., Ferreira M.J.P. (2012). Jacaranone induces apoptosis in melanoma cells via ROS-mediated downregulation of Akt and p38 MAPK activation and displays antitumor activity *in vivo*. PLoS ONE.

